# Please Keep Step

**Published:** 1921-01-15

**Authors:** 


### The Hospital

The Workers' Newspaper of Administrative Medicine and Institutional Life. Administration. National Insurance and Health.

No. 1805. Vol. LXIX. SATURDAY, JANUARY 15, 1921. PRICE TWOPENCE

### PLEASE KEEP STEP.

With the promised appointment of a committee to inquire into the financial position of voluntary hospitals this question may be said to be in a sense *sub judice*, but there has been nothing approaching a lull in public general discussion of hospital topics during the past few weeks. Why there has been so much delay in appointing the Committee has not been disclosed, and a loss of time in a period of great emergency, especially after an assurance had been given that the Committee would be appointed quickly and asked to report an soon as possible, is disquieting to those who appreciate the soundness of Lord Knutsford's warning that the Ides of March, when tradesmen's quarterly accounts are due, will be the critical financial period.

It is, however, too much to look for a complete truce in the matter of argument of hospital politics. All that can be expected is that words should be weighed a little more carefully than in normal times, in order that the presentation of apparently brilliant but really unworkable schemes of reform and amelioration should not discount in any way the findings of the Committee, from which so many thinking hospital people hope to gather substantial help and guidance.

We do not in this connection criticise the discussion at the Harveian Society, based upon the able papers of Dr. C. M. Wilson, Dr. C. Buttar, and Mr. E. W. Morris, which for practical purposes has no more or less significance than the proceedings of an eminent debating society. The formation of a larger and more representative Metropolitan Asylums Board, suggested by Mr. Morris, does great credit to that gentleman's ingenuity and ability, but it has the same tendency, and one which supporters of the voluntary system are not yet ready to adopt, as many other schemes recently suggested—that it bears the hospital State-ward and rate-ward.

The object of the British Medical Association meeting—namely, to determine the attitude of the Association when and if it is represented before the Committee of Inquiry, is sound enough, but one at least of the resolutions passed entered upon deep and dangerous waters which could have, but had not, been explored. We refer to the pound-for-pound idea, or in British Medical Association language " that in times of financial difficulty it is desirable that the Central Government should subsidise voluntary hospitals, and that such subsidies, in the case of any individual hospitals, should take the form of a contribution proportional to the income received by the hospital from voluntary and other contributions."

It is our intention, and we trust our achievement, that this journal should be of help and convenience to medical men and lay institutional workers. If a lay body should pass a resolution on a purely medical matter we should have a word to say and when a medical gathering tackles a purely financial problem we have likewise some observations to make.

We have always expressed our belief that it is to the West the hospital world would better look for guidance than to the East. The great hospitals of the United States with a system under which the rich pay for the poor is one, from the voluntary or charitable standpoint, which is more worthy of emulation than the rapidly changing systems of Australia, whence the equal State contribution theory emanates. In the Commonwealth the voluntary hospital system varies in each State, and is too complicated here to be explained in full. The pound-for-pound method of State help is fairly general, but nobody can claim that it is popular or that through it the hospitals are any better off than they were on a purely voluntary basis. Against donations, subscriptions, and bequests the Government has for many years past provided an equal pound for every pound given with the effect that charitable contributions have decreased, and many of the hospitals have become involved in as great financial difficulties as those in the home country. The Government has a distinct voice in hospital management; the Prince Alfred Hospital, Sydney, to take one of the largest and most important institutions, has five directors out of fifteen who are not elected by the subscribers. We do not say that this is in itself an objectionable feature, but it is one which must be known and appreciated.

Recently the great hospital last mentioned issued an appeal, which obtained widespread support in its progress and backing, but in the end produced very disappointing results. The public are not going to put their contributions side by side with a definite State subsidy, and to help to bring about such a system is to lay the foundation for a State hospital service within a measurable period of time.

It is quite another thing to ask for Government payment for public services in the direction of matters for which the State has already accepted responsibility. A substantial payment towards medical education is a reasonable request, and one of the clearest arguments for its necessity is the proved fact that the teaching hospitals are more expensive to maintain than the general hospitals without schools. Contributions towards the cost of training nurses and for research work are also fair payments towards services, which are not merely, or even largely, for the benefit of hospitals alone. Reasonable dealing with the hospitals as regards the treatment of National Health Insurance patients should have been accorded years ago, and must come without delay from the enormous accumulations of the Approved Societies.

We do not dare to say that the hospital financial outlook is bright, but there are here and there glimmers of light which show that the sun is trying to break through after a dark and cloudy day. Towards the close of the past year many hospitals received a welcome grant from the National Relief Fund, the King Edward's Fund, Christmas contributions were but little affected by the emergency grants of the summer, and everywhere the better-organised workers' contributions are promising well, in spite of widespread unemployment. Altogether the prospect is much better than could have been hoped for six months ago, and we sincerely trust that what is taking the form of a national understanding of hospital problems and difficulties will not be prejudiced by premature or ill-considered schemes of change without betterment.

